# Cell Mapping Toolkit: an end-to-end pipeline for mapping subcellular organization

**DOI:** 10.1093/bioinformatics/btaf205

**Published:** 2025-06-09

**Authors:** Joanna Lenkiewicz, Christopher Churas, Mengzhou Hu, Gege Qian, Mayank Jain, Maxwell Adam Levinson, Sadnan Al Manir, Yue Qin, Dylan Fong, Keiichiro Ono, Jing Chen, Chengzhan Gao, Dexter Pratt, Jillian A Parker, Timothy Clark, Trey Ideker, Leah V Schaffer

**Affiliations:** Department of Medicine, University of California San Diego, La Jolla, CA, 92037, United States; Department of Medicine, University of California San Diego, La Jolla, CA, 92037, United States; Department of Medicine, University of California San Diego, La Jolla, CA, 92037, United States; Department of Medicine, University of California San Diego, La Jolla, CA, 92037, United States; Department of Medicine, University of California San Diego, La Jolla, CA, 92037, United States; Department of Public Health Sciences (Biomedical Informatics), University of Virginia School of Medicine, Charlottesville, VA, 22903, United States; Department of Public Health Sciences (Biomedical Informatics), University of Virginia School of Medicine, Charlottesville, VA, 22903, United States; Department of Medicine, University of California San Diego, La Jolla, CA, 92037, United States; Eric and Wendy Schmidt Center, Broad Institute of MIT and Harvard, Boston, MA, 02142, United States; Department of Medicine, University of California San Diego, La Jolla, CA, 92037, United States; Department of Medicine, University of California San Diego, La Jolla, CA, 92037, United States; Department of Medicine, University of California San Diego, La Jolla, CA, 92037, United States; Department of Medicine, University of California San Diego, La Jolla, CA, 92037, United States; Department of Medicine, University of California San Diego, La Jolla, CA, 92037, United States; Department of Medicine, University of California San Diego, La Jolla, CA, 92037, United States; Department of Public Health Sciences (Biomedical Informatics), University of Virginia School of Medicine, Charlottesville, VA, 22903, United States; Center for Advanced Medical Analytics, University of Virginia School of Medicine, Charlottesville, VA, 22903, United States; University of Virginia School of Data Science, Charlottesville, VA, 22903, United States; Department of Medicine, University of California San Diego, La Jolla, CA, 92037, United States; Department of Computer Science and Engineering, University of California San Diego, La Jolla, CA, 92037, United States; Department of Bioengineering, University of California San Diego, La Jolla, CA, 92037, United States; Department of Medicine, University of California San Diego, La Jolla, CA, 92037, United States

## Abstract

**Summary:**

Cells are organized as a hierarchy of macromolecular assemblies, ranging from small protein complexes to entire organelles. Various technologies have been developed to elucidate subcellular architecture at different scales, such as mass spectrometry approaches for mapping protein biophysical interactions and immunofluorescence imaging for mapping protein localization. We present the Cell Mapping Toolkit, which is designed to systematically integrate data from different modalities into unified hierarchical maps of subcellular organization. The toolkit facilitates an end-to-end pipeline including processing datasets, integrating modalities, and visualizing the final cell map with rich metadata including provenance documentation at each step. The Cell Mapping Toolkit provides researchers with tools for analyzing, integrating, and visualizing diverse protein datasets in a robust and reproducible framework.

**Availability and implementation:**

The code is freely available and is hosted on GitHub at https://github.com/idekerlab/cellmaps_pipeline. Comprehensive documentation and practical examples are provided at https://cellmaps-pipeline.readthedocs.io/.

## 1 Introduction

A fundamental goal in biology is mapping protein assemblies and their spatial distribution within cells, with downstream applications including understanding disease phenotypes, revealing drug targets, and interpreting genetics (Karr *et al.* 2012, Johnson *et al.* 2023, Cesnik *et al.* 2024). Various technologies currently exist for mapping biological systems, each measuring different biological scales ranging from nanometers to microns (Wilhelm *et al.* 2014, Mulvey *et al.* 2017, Thul and Lindskog 2018, Luck *et al.* 2020, Richards *et al.* 2021, Skinnider *et al.* 2021, Reed *et al.* 2024). For example, approaches including affinity purification coupled with mass spectrometry (AP-MS) (Choi *et al.* 2012, Huttlin *et al.* 2015, 2021, Gordon *et al.* 2020) or size exclusion chromatography mass spectrometry (SEC-MS) (Havugimana *et al.* 2012, Bludau *et al.* 2020, Fossati *et al.* 2023) enable the identification of protein-protein interactions (PPIs) and protein complexes. At larger biological scales, approaches including subcellular fractionation (Dunkley *et al.* 2004, Mulvey *et al.* 2017) and immunofluorescence (IF) (Thul *et al.* 2017) or endogenous fluorescent-tagged imaging (Cho *et al.* 2022) determine the specific localization of proteins within larger cell compartments. There are also approaches for mapping protein functional associations, such as genome-wide CRISPR perturbations (Dixit *et al.* 2016, Replogle *et al.* 2022) that determine pairs of proteins with similar transcriptional effects upon knockdown.

These technologies have typically been applied separately, each revealing different information about protein organization and with unique advantages and challenges (Christopher *et al.* 2021, Richards *et al.* 2021). Integrating data from multiple protein mapping technologies presents an opportunity to generate a more comprehensive understanding of subcellular structure. Toward this goal, we recently developed an approach for integrating diverse data modalities into a hierarchical map of protein assemblies (Qin *et al.* 2021, Schaffer *et al.* 2025), robustly revealing more protein assemblies in the cell than any individual dataset alone. We developed the Cell Mapping Toolkit to streamline and productionize this process of integrating datasets into hierarchical cell maps and to make the tools accessible to a broad research community. The toolkit is a scalable and user-friendly software tool consisting of a set of Python packages. In what follows, we describe the Cell Mapping Toolkit and present a tutorial demonstrating its application to currently available datasets.

## 2 Software implementation

**Figure 1.**
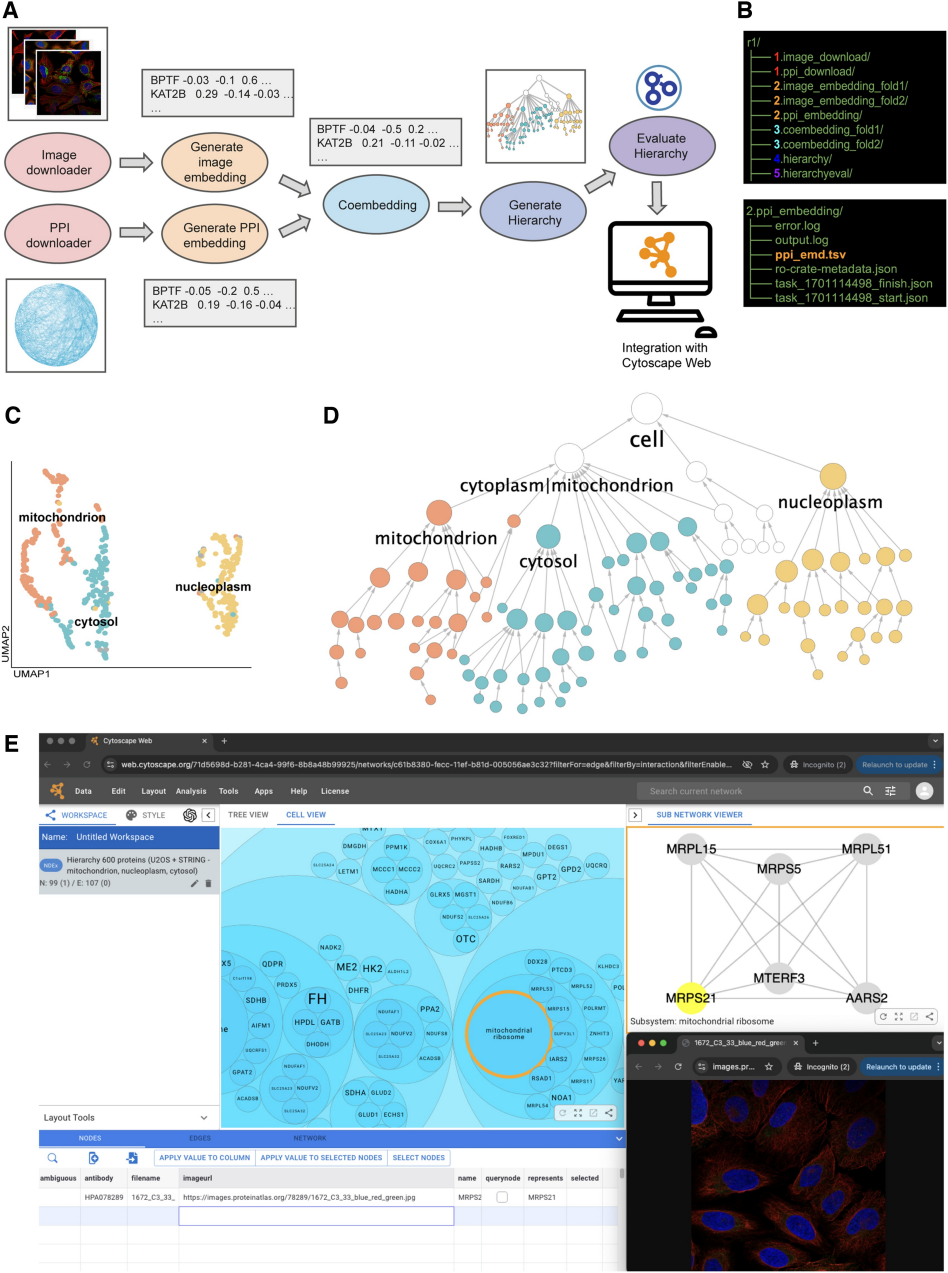
Demonstration of the Cell Mapping Toolkit. (A) Overview of processing steps in Cell Mapping Toolkit. (B) Directory structure and outputs of the Cell Mapping Toolkit. The upper panel displays all output directories created after running the full pipeline, with each folder corresponding to a specific step in the process. The lower panel shows the resulting files in the directory generated by the PPI embedding step. These include log files, an embedding file, and a RO-Crate metadata file capturing provenance information. (C) Multimodal embedding of proteins based on integration of AP-MS and imaging data, reduced to two dimensions using the UMAP method. (D) Proof-of-concept cell map generated with 600 proteins using associations in STRING and images from the HPA. The hierarchy is represented in a tree view. The size of nodes is proportional to the number of proteins. Nodes are colored based on subcellular location, as defined by HPA. (E) Cell map in cell view (circle packing) on Cytoscape Web. Selecting a protein assembly cluster in the cell map shows underlying interaction data and links to the images.

The Cell Mapping Toolkit comprises a set of Python packages that are pip installable and facilitate data downloading, processing, co-embedding, and cell map hierarchy generation and evaluation (Fig. 1A). The toolkit provides auto-generated documentation hosted on ReadTheDocs, includes unit testing that runs automatically on code commits, and adheres to a strict version control policy to minimize integration issues.

The main architecture follows a pattern where each step creates a directory on the filesystem that stores one or more data files (Fig. 1B). Subsequent steps use these data files, and each directory is registered as an RO-crate (Soiland-Reyes *et al.* 2022) via FAIRSCAPE framework (Levinson *et al.* 2022) for provenance. As part of the RO-crate, each tool registers the code used to generate the data, as well as required provenance information for any imported data. This provenance and metadata ensure that every step implemented with the toolkit is documented and reproducible, which is important for downstream analysis and interpretability of cell maps (Wilson *et al.* 2021, Clark *et al.* 2024). Schemas defining the format for each file are available at Zenodo (https://doi.org/10.5281/zenodo.14200177). The organization of the toolkit enables users to substitute any step with external code and new methods, as long as the output matches the required format specified by each step. Each tool in the pipeline has a command line interface, as well as a programmatic interface that can be called individually or as a whole. Here, we describe each step in the cell mapping process and the associated tool in the Cell Mapping Toolkit.

### 2.1 Step 1: Image and Protein-Protein interaction data downloaders 

The download process is managed by scripts that ensure the data is fetched, followed by validation against predefined schemas and packaging with rich metadata including provenance into standard RO-Crate packages by the FAIRSCAPE client. We developed an Image Data Downloader, which currently supports downloads from the Human Protein Atlas (HPA) (Thul and Lindskog 2018) using a .tsv file that specifies the required images or a text file with a list of proteins. We also created a PPI Data Downloader, which formats gene names and attributes for an input edge list.

### 2.2 Step 2: Embed each data modality

We generated tools to create embeddings (a low-dimensional representation extracted from complex high-dimensional input) for each data modality, implementing algorithms to support image and network-based data. For images, the default embedding is the penultimate layer of an HPA image classification model [densenet (Ouyang *et al.* 2019)] which captures information about protein subcellular localization. For network-based data modalities, we developed a PPI Embedding tool that runs the node2vec (Grover and Leskovec 2016) algorithm on the network, which generates an embedding for each node (i.e. protein) that captures relative relationships about the interaction neighborhoods.

### 2.3 Step 3: Co-embed the data modalities

The embeddings for each data modality—generated either by our toolkit for image and network embeddings or externally for other data types—are integrated using the co-embedding tool (Schaffer *et al.* 2025). The integration uses a self-supervised learning approach (Bao *et al.* 2022) to learn a unified embedding for each protein. The toolkit provides utilities for evaluating the co-embeddings, including assessing the similarities of protein pairs present in known complexes and visualizing the embedding space using the UMAP method (McInnes *et al.* 2018) (Fig. 1C).

### 2.4 Step 4: Generate hierarchy of protein assemblies

Hierarchy generation within the toolkit begins by calculating cosine similarities of the co-embedding between each protein pair. A set of protein-protein similarity networks is generated at various thresholds, and pan-resolution community detection is performed using Hierarchical community Decoding Framework (Zheng *et al.* 2021) to generate a multi-scale hierarchy.

### 2.5 Step 5: Evaluate the hierarchy

The hierarchy is evaluated for overlap with documented protein assemblies using multiple resources including Gene Ontology (Ashburner *et al.* 2000, Aleksander *et al.* 2023) cellular component terms, the comprehensive resource of mammalian protein complexes [CORUM (Tsitsiridis *et al.* 2023)], and HPA cellular compartments. Additionally, the toolkit provides the option to annotate assemblies in the cell maps using a large language model (LLM) approach to name sets of proteins and assign a name confidence score using a designed prompt (Hu *et al.* 2023). The final annotated hierarchy is saved in a format allowing visualization in Cytoscape, and can be uploaded to the Network Data Exchange (NDEx, https://www.ndexbio.org/) for storage, sharing, manipulation, and publication (Pratt *et al.* 2015, Pillich *et al.* 2021). Finally, the toolkit provides documentation and utilities to assess the robustness of protein assemblies across multiple jackknife resamplings.

## 3 Results

### 3.1 Environmental setup

The Cell Maps Pipeline Python package can be installed using the following command: pip install cellmaps_pipeline.

This package is compatible with Python versions 3.8 through 3.11. For optimal performance and isolation of dependencies, it is strongly recommended to utilize an Anaconda environment (docs.anaconda.com).

### 3.2 Data acquisition

To create a proof-of-concept cell map, we randomly selected a set of 600 proteins, including 200 localized to each of three different cell compartments (nucleoplasm, mitochondria, and cytosol), as defined by HPA. A list of these proteins was provided as an input in the Image Downloader to obtain images (see below). Protein-protein Interactions (PPIs) were obtained from the high-confidence (score ≥ 0.7) STRING (Snel *et al.* 2000, Szklarczyk *et al.* 2019) interactome. We selected a subnetwork for the same 600 proteins from the network on NDEx (https://ndexbio.org/, uuid: 24823fd3-6ebb-11ef-a7fd-005056ae23aa), saved as an edgelist in a .tsv file.

### 3.3 Data provenance

A provenance file detailing the information about input data must be provided to adhere to FAIR principles. Users have the option to generate a sample provenance file using the following command:

cellmaps_pipelinecmd.py . ––example_provenance>provenance.json

Once the sample provenance file is generated, the user should edit it to include the necessary information, including name, organization name, project name, cell line, treatment, gene set, and information about individual input files.

### 3.4 Running the cell mapping toolkit

The Cell Mapping Toolkit can be executed by running cellmaps_pipelinecmd.py with required arguments including the output directory, provenance file, and input data.

cellmaps_pipelinecmd.py ./cellmaps_pipeline_outdir [FLAGS WITH PARAMETERS]

Alternatively, individual toolkit steps can be run separately through their respective Python packages.

1. Downloading images from HPA
    cellmaps_imagedownloadercmd.py ./1.image_downloader ––protein_list proteins.txt ––cell_line U2OS ––provenance provenance_images.json
2. Generating embeddings in image and PPI data
    cellmaps_image_embeddingcmd.py ./2.image_embedding ––inputdir ./1.image_downloader
    cellmaps_ppi_embeddingcmd.py ./2.ppi_embedding ––inputdir ./string_ppi_dir ––provenance provenance_ppi.json
3. Integrating the embeddings (co-embedding)
    cellmaps_coembeddingcmd.py ./3.coembedding ––embeddings ./2.ppi_embedding ./2.image_embedding
4. Generating the hierarchical cell map
    cellmaps_generate_hierarchycmd.py ./4.hierarchy ––coembedding_dirs ./3.coembedding
5. Evaluating cell map for known components (Fig. 1D)
    cellmaps_hierarchyevalcmd.py ./5.hierarchyeval ––hierarchy_dir ./4.hierarchy

### 3.5 Visualization and sharing

The Cell Mapping Toolkit can be used to upload the final hierarchy to NDEx, a platform for sharing biological network data (Pratt *et al.* 2015, Pillich *et al.* 2021). NDEx provides other users easy access to the hierarchy, making it accessible to a broader community and facilitating collaboration. Users can upload their hierarchy using the cellmaps_generate_hierarchy tool included in the toolkit, using their credentials to the NDEx account with the following command:

cellmaps_generate_hierarchycmd.py ./5.hierarchyeval --mode ndexsave --ndexuser < USER >

Once the hierarchy is uploaded, a link is generated that allows the user to access and interact with the hierarchy through Cytoscape Web (web.cytoscape.org), a new web application based on the desktop application (Shannon *et al.* 2003, Smoot *et al.* 2011). This platform provides a visual interface where users can explore and interact with the cell map in two views, the tree hierarchy (Fig. 1D) and a cell view (Fig. 1E). Users can also browse the underlying subnetworks and links to view images for each protein assembly.

### 3.6 Cell mapping toolkit test users

As part of the National Institutes of Health (NIH) Bridge2AI program (Clark *et al.* 2024), we have hosted a series of in-person and virtual codefests where users implement and test the Cell Mapping Toolkit. These codefests resulted in a total of approximately 50 participants who ran the toolkit and created cell maps from different sample datasets. We used feedback from the users to fix unexpected issues and improve the documentation and guides. This number of test users highlights the stability of the toolkit on a variety of computational systems and by personnel of varying computational experiences.

## 4 Conclusions

We have developed the Cell Mapping Toolkit to build and analyze hierarchical maps of cell architecture via integration of diverse data modalities. The toolkit’s modularity and flexibility enable users to adapt the pipeline to their specific datasets and applications. The tool is user-friendly, extensible, and ensures the creation of trackable and reproducible results.

## Data Availability

The protein interaction data used for the proof-of-concept cell map are available at https://ndexbio.org under uuid 24823fd3-6ebb-11ef-a7fd-005056ae23aa. Images are available at the Human Protein Atlas (https://www.proteinatlas.org/).
